# Systemic treatments for breast cancer brain metastasis

**DOI:** 10.3389/fonc.2022.1086821

**Published:** 2023-01-06

**Authors:** Qiuchi Chen, Jie Xiong, Yuxi Ma, Jielin Wei, Cuiwei Liu, Yanxia Zhao

**Affiliations:** Cancer Center, Union Hospital, Tongji Medical College, Huazhong University of Science and Technology, Wuhan, China

**Keywords:** breast cancer, brain metastasis, systemic treatment, targeted treatment, endocrine treatment

## Abstract

Breast cancer (BC) is the most common cancer in females and BC brain metastasis (BCBM) is considered as the second most frequent brain metastasis. Although the advanced treatment has significantly prolonged the survival in BC patients, the prognosis of BCBM is still poor. The management of BCBM remains challenging. Systemic treatments are important to maintain control of central nervous system disease and improve patients’ survival. BCBM medical treatment is a rapidly advancing area of research. With the emergence of new targeted drugs, more options are provided for the treatment of BM. This review features currently available BCBM treatment strategies and outlines novel drugs and ongoing clinical trials that may be available in the future. These treatment strategies are discovered to be more efficacious and potent, and present a paradigm shift in the management of BCBMs.

## 1 Introduction

In 2020, the incidence of breast cancer (BC) surpassed lung cancer and was ranked first, accounting for 11.7% of all cancer, with about 2.3 million new cases, worldwide (1). With the BC treatment development, the frequency of central nervous system (CNS) metastases has steadily increased because BC patients survive long enough to be at risk of developing CNS metastasis (2). About 7% of metastatic BC will develop brain metastases (BMs) (3), including parenchymal and leptomeningeal disease, accounting for roughly 17% of all BMs, and the second major cause of BMs after lung cancer (4, 5). Risk of BM is variable across BC subtypes. BM is majorly observed in triple-negative BC (TNBC) and human epidermal growth factor receptor 2 (HER2)-positive BC. In comparison with the hormone receptors (HR) positive subtype, the risk of BM development in HER2 positive and TNBC is 2-5 times higher (6, 7). Although the advanced BC treatment has remarkably prolonged the patient’s survival, the prognosis of BC brain metastasis (BCBM) is still substandard. The median patients’ survival in parenchymal and leptomeningeal disease was 3 to 23 and 3 to 4 months, respectively (8, 9). The survival time for HER2+ patients is reported to be the longest, while that of TNBC patients is considered the least (10). In a large multi-center study, the median overall survival (OS) after the diagnosis of BCBM with HR+/HER2+ was 18.9 months, with HR–/HER2+ was 13.1 months, with HR+/HER2– was 7.1 months and for triple-negative was 4.4 months (11). Due to the substandard prognosis and lack of efficient treatment strategies, BCBM represents a unique and challenging clinical problem.

The brain is the most complex and unique organ in the body, and because of this, there are differences in treatment. The brain microenvironment is composed of two different components, parenchyma and leptomeninges (12). The brain parenchyma comprises cells that are not present anywhere else in the body, which include astrocytes, oligodendrocytes, microglia, and neuronal cells. The leptomeninges is mainly filled with the circulating cerebrospinal fluid (CSF) produced by the choroid plexus (13). The blood-brain barrier (BBB) is the main gatekeeper of the CNS (13). BBB is a unique neurovascular unit consisting of a continuous segment of non-porous blood vessels and interacts with parietal, immune, glial, and nerve cells to carefully modulate the movement of ions, molecules, and cells between the brain and the blood (14, 15), thereby protecting the CNS from pathogens, toxins, injury, inflammation, and diseases, while also providing a barrier for drugs delivery to the brain (16). Another important factor that produces drug resistance during BM treatment is the BBB efflux transporter systems, which reversely transport and prevent drug penetration into CNS. These include P-glycoprotein, BC-resistance protein, multidrug resistance-associated proteins, etc. (17). The formation of metastatic tumors may partially disrupt the BBB, allowing it to become more permeable. However, it is still not sufficiently or homogeneously permeable for effective drug therapy (18).

**Figure 1:**
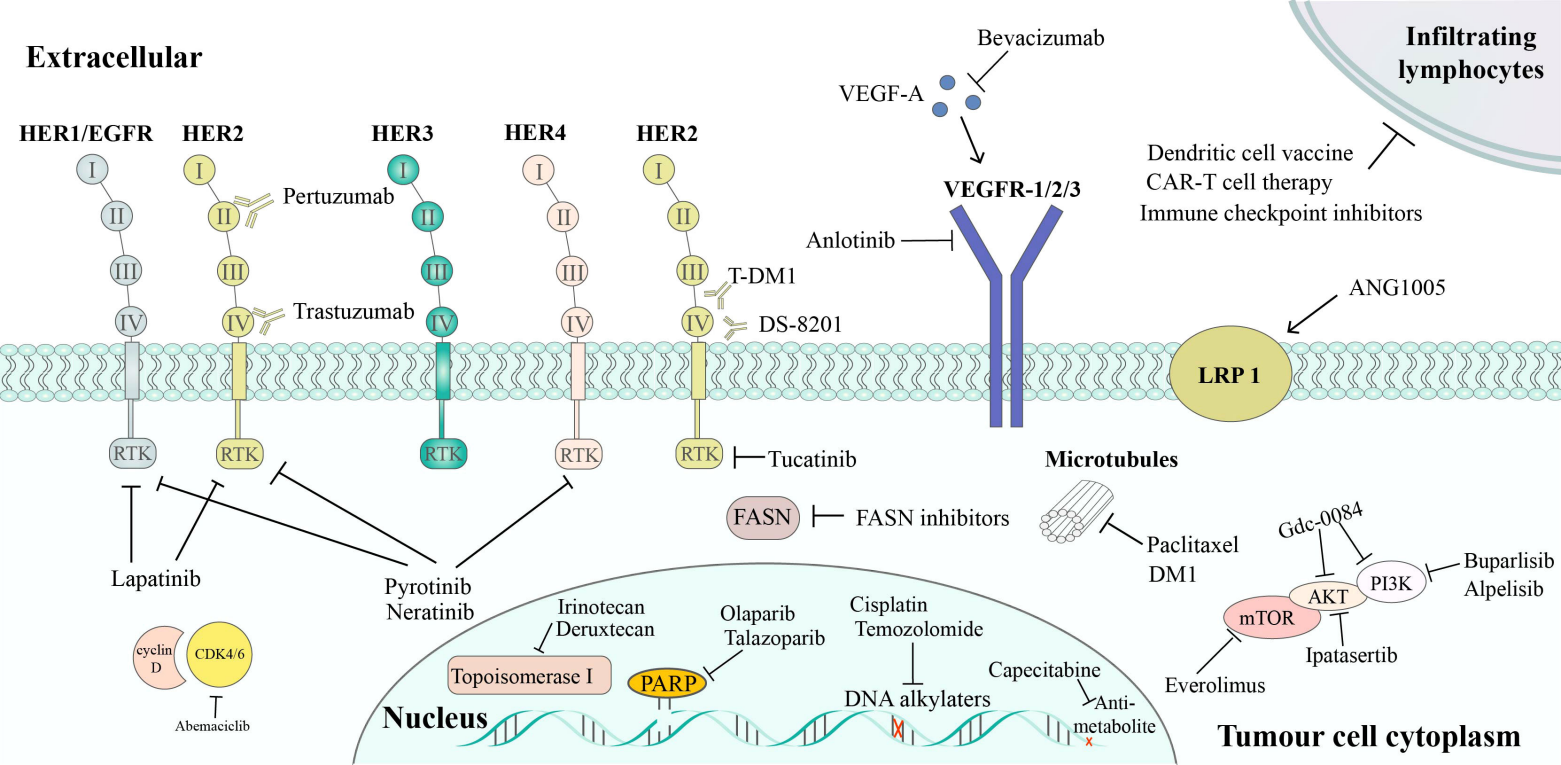
Targeted therapy of BCBM treatment. HER2 inhibitors includes: trastuzumab, which binds to subdomain IV of HER2, leading to the inhibition of HER2 signaling; pertuzumab, which binds to subdomain II of HER2, preventing HER2/HER3 dimerization; T-DM1, an ADC of trastuzumab and the cytotoxic agent DM1; DS-8201, another ADC that combines trastuzumab and deruxtecan, a potent topoisomerase I inhibitor; lapatinib, which is a reversible TKI of HER1 and HER2; tucatinib, a specific HER2 TKI; pyrotinib and neratinib, TKIs that inhibits HER1, HER2, and HER4. Pathway inhibitors include: PI3K/Akt/mTOR pathway inhibitors (buparlisib, alpelisib, Gdc-0084, ipatasertib, and everolimus); CDK4/6 inhibitors (abemaciclib), VEGF pathway inhibitors (anlotinib, bevacizumab). Other therapies include: PARP inhibitors, FASN inhibitors, chemotherapy, and immunotherapy.

Currently, the main treatment strategies for BCBM include local (surgical resection and radiotherapy) and systemic treatments (19). In the study by Minniti et al, it has been demonstrated that BM patients who received multifraction (3× 9 Gy) stereotactic radiosurgery after surgery had an improved local control rate of 91% at 12 months (20). Systemic treatments are important for controlling CNS diseases and improving patients’ survival (19). Although heavy literatures on BBB’s effect and anti-cancer drugs’ efficacy on BCBM is deficient, our knowledge about the effect of systemic treatments on BCBM is rapidly increasing. This review focuses on the currently available system treatment drugs for BCBM, such as chemotherapy, target therapy, endocrine therapy, immunotherapy, and novel therapies that may be available in the future (**Figure 1**).

## 2 Treatment for HER2-Positive BCBM

HER2+ BC accounts for approximately 20% of all BC cases (21). HER is a group of reversible tyrosine kinase receptors, comprising four members: epidermal growth factor receptor (EGFR)/HER1, HER2, HER3, and HER4. These have crucial functions in tumorigenesis and help tumor cells escape anti-tumor immunity. Anti-HER2-targeting drugs can inhibit the kinase activity of these HER receptors and prevent further cancer cell survival and proliferation (22). HER2-targeting drugs involve tyrosine kinase inhibitors (TKIs), monoclonal antibodies, and antibody-drug conjugates (ADCs). TKI is a small molecule and comprises lapatinib, neratinib, tukatinib, etc. have a considerably lower molecular weight, allowing them a more efficacious penetration through the BBB (21). **Table 1** lists the characteristics of studies reporting on outcomes related to BMs in patients with HER2-positive BC.

**Table 1:** Characteristics of studies reporting on outcomes related to BMs in patients with HER2-positive BC.

Anti-HER2 Agent	Authors	Study	Population	Outcomes related to BM
Trastuzumab	Brufsky et al. (23)	Prospective	Newly diagnosed HER2+ MBC	Time to BM progression, OS
Trastuzumab	Olson et al. (24)	Retrospective	HER2+ BC	Incidence of BM as first metastatic site
Pertuzumab + high dose trastuzumab	Lin et al. (25)	Prospective	HER2+ BCBM after RT	CNS ORR
Trastuzumab + pertuzumab + taxane	Gamucci et al. (26)	Retrospective	HER2+ MBC	ORR, PFS, OS
Pertuzumab + capecitabine + trastuzumab	Urruticoechea et al. (27)	Prospective	HER2+ MBC	PFS, OS
Lapatinib	Lin et al. (28)	Prospective	HER2+ BCBM, prior trastuzumab and RT	CNS ORR, PFS, OS
Lapatinib + capecitabine	Bachelot et al. (29)	Prospective	HER2+ BC with untreated BM	CNS ORR, PFS
Lapatinib + WBRT	Lin et al. (30)	Prospective	HER2+ BCBM	Maximum tolerated dose, CNS ORR, PFS, OS
Neratinib +capecitabine	Freedman et al. (31)	Prospective	HER2+ BCBM	CNS ORR, PFS, OS
Neratinib + paclitaxel	Awada et al. (32)	Prospective	HER2+ MBC	Incidence BM, Time to BM, Time to BM progression
Pyrotinib + capecitabine	Yan et al. (33)	Prospective	HER2+ BCBM	CNS ORR, the time to CNS response, PFS, OS
Tucatinib + trastuzumab + capecitabine	Murthy et al. (34)	Prospective	HER2+ MBC, prior treated with trastuzumab, pertuzumab, and trastuzumab emtansine	PFS, OS, ORR
T-DM1	Krop et al. (35)	Retrospective	HER2+ MBC	Incidence of BM, PFS, OS
T-DM1	Montemurro et al. (36)	Prospective	HER2+ BCBM	CNS ORR, PFS, OS
DS-8201	Modi et al. (37)	Prospective	HER2+ MBC, previous treated with T-DM1	PFS
DS-8201	Cortés et al. (38)	Prospective	HER2+ MBC	PFS, OS, ORR

BC, breast cancer; BCBM, breast cancer brain metastases; BM, brain metastasis; CNS, central nervous system; DS-8201, trastuzumab deruxtecan; HER2, human epidermal growth factor receptor 2; MBC, metastatic breast cancer; OS, overall survival; ORR, objective response rate; PFS, progression-free survival; RT, radiotherapy; T-DM1, trastuzumab emtansine; WBRT, whole brain radiotherapy.

Monoclonal antibody drug, such as trastuzumab, was the first authorized targeted therapy against HER2+ BC. In the registHER study, trastuzumab treatment after CNS metastatic diagnosis significantly improved OS statistically (treatment vs. no trastuzumab treatment, 17.5 vs. 3.8 months) (23). Trastuzumab also has inconsistent responses in intracranial and extra-cranial tumors, as it cannot entirely cross the BBB to play a preventive role (39). A meta-analysis of HER2+ BC patients (n=9020) showed that CNS metastasis accounted for a greater proportion of the first relapse site in patients receiving trastuzumab treatment (24). Among HER2+ patients receiving trastuzumab adjuvant therapy, the CNS metastasis incidence as the first site of disease recurrence was 2.56% (95% CI 2.07%-3.01%) than in those who did not receive adjuvant trastuzumab 1.94% (95% CI 1.54%-2.38%) (24). Pertuzumab acts on a different HER2 site, and it prevents HER2/HER3 dimerization (40) which can lead to significant PI3K-Akt activation (main signaling pathway in BC cells) (41). When combined with trastuzumab, it can play a complementary role and provides efficient treatment for HER2+ BC patients. However, this dual-target therapy fails to show an advantage in BMs (42). In the phase II PATRICIA investigation, the CNS ORR for pertuzumab plus high-dose trastuzumab was 11% (95% CI 3-25) (25). In early studies, the trastuzumab–pertuzumab dual-target therapy combined with other agents indicated some activity in BCBM (26, 27, 43). A retrospective observational investigation which included 264 HER2+ BCBM patients revealed that dual HER2-blockade and taxanes treatment produced an ORR of 52.4% in baseline BM patients (26). In the phase III PHEREXA trial, after pertuzumab was added to capecitabine + trastuzumab, the PFS markedly elevated in the subgroup of HER2+ baseline BM patients (n = 53; HR 0.29, 95% CI 0.15–0.60) (27). All in all, trastuzumab and partuzumab, as macromolecular monoclonal antibodies, have limited their ability to pass the blood-brain barrier, but their combination with other chemotherapy drugs has increased their intracranial activity to some extent.

Lapatinib inhibits EGFR and HER2 and is authorized to be given with capecitabine as a combined therapy for metastatic BC (44). The CNS ORR of mono lapatinib therapy is approximately only 6% (28), while the CNS ORR of lapatinib + capecitabine in 13 BCBM patients who prior received trastuzumab and radiation therapy is 38% (95% CI, 13.9-68.4) (45). At the same time, a single-arm phase II clinical trial showed that in untreated BCBM patients, the CNS ORR of lapatinib + capecitabine could reach 65.9% (29). Lapatinib + capecitabine is also associated with less incidence of CNS at the first progression. A phase III randomized trial indicated that in comparison with capecitabine monotherapy, the CNS involvement cases at first progression were fewer (2% vs 6%, P = 0.045) in combined therapy (46). Lapatinib combined with fractionated radiotherapy may be useful against HER2+ BCBM in the tumor xenograft model (47). In a Phase I investigation, lapatinib with WBRT had a higher CNS ORR (79%) than that of WBRT alone (36%) in 28 BCBM patients (30).

Neratinib irreversibly inhibits HER2, HER1, and HER4 (48), and may pass through the intact BBB by inhibiting ATP-binding cassette B1 transport function which is one of the dominant efflux transporters in the BBB (49, 50). In a phase II trial of neratinib combined with capecitabine treatment in refractory HER2+ BCBM patients, 33% and 49% of the patients with and without previous lapatinib treatment achieved CNS ORR, respectively (31). In a randomized clinical trial of previously untreated metastatic HER2+ BC, 8.3% and 17% of patients in the neratinib + paclitaxel and trastuzumab + paclitaxel groups experienced symptomatic or progressive CNS recurrence, respectively (32), demonstrating its preventive effect on the CNS metastasis of BC.

Pyrotinib is an irreversible TKI that targets HER1, HER2, and HER4 (51). A phase II clinical trial (PERMEATE trial) showed the intracranial ORR was 74.6% in a radiation-naive population with pirotinib + capecitabine (cohort A) (n=59) and 42.1% in cohort B (n=19) included patients who had progressive disease after radiotherapy (33). The median PFS in cohort A was 11.3 months and 5.6 months in cohort B (33). PERMEATE research adds strong medical evidence for drug treatment of BM, especially for patients with new BM. However, the efficacy of this regimen still needs more randomized controlled trials for further verification.

Tucatinib is a selective HER2-targeting TKI, and has fewer side effects than other TKIs due to its lack of strong EGFR inhibition (10). In 2020, Tucatinib is authorized by the Food and Drug Administration (FDA) for HER2+ BCBM treatment. A randomized clinical trial (HER2CLIMB trail) was conducted where tucatinib was given with trastuzumab+ capecitabine as a combination therapy to patients who were initially treated for HER2+ metastasis BC. In BM patients, the estimated 1-year PFS in the tucatinib and placebo groups was 24.9% (95% CI, 16.5 to 34.3) and 0%, respectively. The median PFS in the tucatinib and placebo groups was 7.6 months (95% CI 6.2-9.5) and 5.4 months (95% CI 4.1-5.7), respectively. The CNS ORR was 40.6% (95% CI 35.3-46.0) in tucatinib group compared with 22.8% (95% CI 16.7-29.8) in placebo group (P<0.001). However, the side effects such as the incidences of diarrhea and hepatic injury (grade 3 or higher) were more frequent than those in the control group (34).

Trastuzumab emtansine (T-DM1), an ADC of trastuzumab and the cytotoxic drug emtansine, a maytansine derivative and microtubule inhibitor, has been aproved for the treatment of HER2+ metastatic BC patients after taxane and trastuzumab therapy, and for the adjuvant therapy of early BC HER2+ patients with the residual invasive disease after neo-adjuvant taxane and trastuzumab therapy (43, 52). In the EMILIA study, the patients with baseline CNS metastases had OS of 26.8 months in the T-DM1 arm, compared with 12.9 months in the capecitabine + lapatinib arm (HR=0.38, P=0.008). PFS in the two treatment arms was similar (5.9 vs. 5.7 months; P=1.0) (35). In a KAMILLA single-arm research, in the 126 measurable BCBM patients, CNS ORR was 21.4% (95% CI 14.6–29.6), whereas, in the 398 baselines BCBM patients, the median PFS and OS were 5.5 months (95% CI 5.3–5.6) and 18.9 (95% CI 17.1–21.3), respectively (36). In short, T-DM1 may be effective in treating HER2+ intracranial lesions.

Another ADC is trastuzumab deruxtecan (DS-8201), comprising a human HER2 antibody, a new enzyme-cleavable linker, and a topoisomerase I inhibitor payload. Compared with T-DM1, the antibody-drug ratio of DS-8201 is higher (8 vs 3–4) (37). The DESTINY-Breast01 trial, evaluating DS-8201 in HER2+ metastatic BC patients with previously treated with T-DM1, revealed a median PFS of 18.1 months (95% CI 6.7–18.1) in the BM subgroup (37). In DESTINY-Breast03, in the 82 BCBM patients, DS-8201 had better efficiency than T-DM1. The median PFS was 15.0 months (95%CI 12.5-22.2) in the DS-8201 group and 3.0 months (95%CI: 2.8-5.8) in the T-DM1 group (HR=0.25; 95%CI 0.31-0.45), and the ORR of DS-8201 group and T-DM1 group were 67.4% and 20.5%, respectively (38, 53).

To summarize, the available data indicate remarkable intracranial efficacy from treatments for HER2-positive BCBM, such as TKIs, monoclonal antibodies, and ADCs. However, some problems need to be solved, for example, treatment-induced diarrhea and hepatic injury may be limiting factors and require appropriate surveillance, and the optimal administration time and sequence of each agent remain uncertain.

## 3 Treatment for HR-Positive BCBM

The patients suffering from HR+ BC are less likely to develop BMs than patients with other subtypes of BC (6). The effects of hormone therapy specifically on BMs are still unclear (54). Concentrations of tamoxifen and its metabolites in the BM tumor and brain tissue were 46 times higher than those in serum, suggesting that tamoxifen might be clinically beneficial (55). Furthermore, in preclinical models, estrogen promoted BMs by altering polarity and suppressing the phagocytic activity of M2 microglia, whereas tamoxifen blocked its polarization and enhanced its phagocytic ability, thereby inhibiting BMs (56). Other hormone therapies, such as megestrol acetate and letrozole, only a small number of case reports have documented the responses of hormonal therapy in BCBM patients, and the efficacy has not been confirmed in large-sample clinical trials (54, 57–60).

The growth of HR+ BC cells depends on cyclin D1, which activates cyclin-dependent kinases 4 and 6 (CDK4 and CDK6), thereby inducing the G1-S phase transition and entering the cell cycle. CDK4/6 inhibitors are reliable treatment options for HR+ BC patients with extra-cranial diseases (10). In preclinical models, CDK4/6 inhibitor abemaciclib has better central permeability than other existing CDK4/6 inhibitors (61, 62), which can reach the therapeutic level in human BM (62). In the JPBO study, abemaciclib was given to HR+/HER2- BCBM patients, and the results showed that 25% of patients did achieve clinical benefit (CR, PR, or SD>6 months), but definite intracranial ORR was only 5.6% (63). The real effectiveness of CDK4/6 inhibitors in treating BMs is not verified yet.

Everolimus is a kinase that acts selectively to inhibit the mammalian target of rapamycin (mTOR), and can easily penetrate the CNS of a mouse model (64). However, the intracranial response in a phase II study of everolimus, trastuzumab, and vinorelbine for the treatment of progressive HER2+ BCBM was only 4% (65). But in the phase Ib/II trial assessing the effect of everolimus, lapatinib, and capecitabine combined therapy for HER2+ BCBM, the CNS ORR was 28% (66), highlighting the need for exploring better treatment modalities for everolimus.

## 4 Treatment for Triple-Negative BCBM

### 4.1 Chemotherapy

Lack of specific therapeutic targets, chemotherapy is the major treatment of TNBC. Chemotherapeutic agents used in BC treatment that can cross the blood brain barrier include capecitabine, platinum compounds, and temozolomide (10). But their efficacy is limited (67) and new chemotherapeutic drugs are still being actively explored.

Capecitabine is an inhibitor of thymidylate synthase (the enzyme required for DNA replication in metastatic cancerous cells) and has been traditionally used as a chemotherapeutic drug (68). Some studies showed that capecitabine or its metabolites may penetrate the BBB, and its efficacy in BCBM patients has been reported in the literature (69–71). Chao and colleagues assessed 873 BCBM patients’ data and reported that those who received chemotherapy survived 2.4 to 12.2 months longer than those who did not. Among patients with recurrent CNS cancer, those who received chemotherapy after local BCBM treatment also had a longer brain metastasizing time. The median OS of capecitabine alone in patients with BCBM was 11.8 months (72).

Cisplatin is a platinum-based drug that alkylates DNA by forming platinum-DNA adducts, which damages DNA, arrest G1/S phase, and promote apoptosis (73). In the in-vitro BBB model, cisplatin at concentrations from 5 µM to 20 µM was shown to reduce the chance of BMs in MDA-MB-231 cells (p<0.05) (74). A phase II investigation comprising 12 BCBM participants revealed that the CNS objective response rate (ORR) of bevacizumab, etoposide, and cisplatin was 75% (95%CI 42.8-94.5) and the median CNS progression-free survival (PFS) was 6.6 months (95% CI 0.8-12.4) (75).

Temozolomide is an oral alkylating agent and has the potential to transport across BBB. It is commonly used for glioma treatment and its efficacy in BC is not clear. However, recent large-scale clinical trials indicated that temozolomide alone or combined with radiotherapy has no clinical advantage (76). In a phase II clinical trial, temozolomide showed no objective responses in 18 patients with observable lesions who previously received extensive treatment (77). In a phase II investigation of whole-brain radiation therapy (WBRT) with or without concurrent temozolomide treatment for BCBM, WBRT combined temozolomide was not better than the WBRT group in ORR, PFS, and OS. At 6 weeks, ORRs for the WBRT arm were 36% and for the WBRT + temozolomide, the ORR was 30%. The median PFS and OS were 7.4 and 11.1 months in the WBRT arm, and 6.9 and 9.4 months in the WBRT + temozolomide arm, respectively (78).

Etirinotecan pegol (NKTR 102) is a four-armed polyethylene glycol polymer, with each arm ending with an irinotecan molecule (79). In its preclinical studies, compared to conventional irinotecan, NKTR 102 treatment can improve the survival rate of the TNBC brain metastatic model (80). In the BEACON trial (a phase III trial), BCBM patients who received NKTR-102 treatment had a markedly lowered death risk than those who received treatment of physician’s choice (TPC) (HR 0.51; P < 0.01), with median OS of 10.0 and 4.8 months, respectively (81). In ATTAIN trial, a phase III study of NKTR 102 versus TPC in metastatic BC patients (82), the median PFS in BM patients of NKTR 102 and TPC was 3.9 and 3.3 months, respectively (HR, 0.59; 95% CI, 0.33-1.05; P =0.07), and the median OS in both the groups was nearly the same (7.8 months for NKTR 102, and 7.5 months for TPC group; HR=0.90; 95% CI, 0.61-1.33; P =0 .60) (83). The ATTAIN randomized clinical trial was not consistent with the positive OS benefit observed in BEACON trial (81, 83).

Sacituzumab govitecan (SG) is an ADC comprising an anti-trophoblast cell-surface antigen 2 (Trop-2) antibody bound with SN-38, an active topoisomerase I inhibitor irinotecan metabolite, capable of crossing BBB (84, 85). Trop-2 is a transmembrane calcium signal transducer greatly expressed in BC (85). In a phase III ASCENT investigation of SG versus TPC for metastatic TNBC, the subgroup assessment of stable BMs patients (n=61) revealed that the median PFS and OS for SG were 2.8 and 6.8 months while these were 1.6 and 7.5 months for TPC, respectively. ORR for SG and TPC was 3% and 0%, respectively (86). Suggesting that about ORR and PFS, SG was better than TPC but not OS.

A new taxane derivative, ANG1005 comprises 3 paclitaxel molecules that are covalently bound with Angiopep-2. It can pass through the BBB and enter the malignant cells by lipoprotein receptor-related protein 1 transport system. ANG1005 has shown a significant CNS efficiency in a phase II clinical trial with an overall intracranial ORR of 15%, intracranial clinical benefit rate (CBR) of 68% in all BCBM patients. The median intracranial PFS and OS were 2.8 months and 7.8 months (87).

In a word, the efficacy of single chemotherapeutic drug was unsatisfactory, and the efficacy of combined chemotherapy was objective (**Table 2**).

**Table 2:** Efficacy of combined chemotherapy trials in BCBM.

Authors	Study	Patients	Treatment	ORR (%)	PFS	OS
Franciosi et al. (88)	Prospective	56 BCBM	Cisplatin + etoposide	38%	/	7.1 months
Christodoulou et al. (89)	Prospective	6 BCBM	Cisplatin + temozolomide	/	2.9 months	5.5 months
Erten et al. (90)	Retrospective	6 TN BCBM	Cisplatin + gemcitabine	66.6%	5.6 months	/
Philippe et al. (91)	Prospective	25 BCBM	Cisplatin + vinorelbine + WBRT	44%	3.7 months	6.7 months
Anders et al. (92)	Prospective	34 TN BCBM	Iniparib + irinotecan	12%	2.14 months	7.8 months
Mehta et al. (93)	Prospective	25 BCBM	Veliparib + WBRT	41%	/	7.7 months

BCBM, breast cancer brain metastases; OS, overall survival; ORR, objective response rate; PFS, progression-free survival; TN, triple negative; WBRT, whole brain radiotherapy.

### 4.2 Vascular Endothelial Growth Factor -A-targeting Monoclonal Antibody

Brain metastatic tumors can utilize the host’s vascular system to make blood vessels abnormal and torturing. Angiogenesis inhibitors can remodel and normalize tumor vascular and can play a crucial part in the treatment of BMs (94). In preclinical mouse models of BCBMs, the vascular endothelial growth factor (VEGF) -A-targeting monoclonal antibody bevacizumab, have been associated with improved OS (94, 95). Two phase II clinical trials have investigated bevacizumab combined with platinum chemotherapy in patients with BCBMs that have progressed after WBRT, with CNS ORR of 63-77% and median OS of 10.5-14.1 months (96, 97). Additionally, bevacizumab also controls intracranial edema. According to a retrospective research, bevacizumab’s edema control rate in the BCBM group was 77.14% (98).

### 4.3 Poly (ADP-ribose) polymerase inhibitors

Homologous recombination is an error-free mechanism for repairing double-strand DNA breaks, and poly (ADP-ribose) polymerase (PARP) inhibitors promote apoptosis of tumor cells by inhibiting DNA homologous recombination. Mutations in the BC susceptibility gene 1 (BRCA1) or BC susceptibility gene 2 (BRCA2), make tumors sensitive to PARP inhibitor therapy (99, 100). About half of patients with BRCA1 or BRCA2 mutations develop BMs in the late stage of BC (101). Olaparib and talazoparib are the PARP inhibitors that are authorized for the treatment of brca1 and brca2-related metastatic BC (10). In the phase III EMBRACA clinical trial, talazoparib notably improved PFS and ORR than that in the TPC group. In the subgroup of BM patients, the benefit of PFS was even higher than that of patients without BM (HR 0.32 vs. HR 0.58) (102), which suggested its possible effect on BCBM.

## 5 Potential strategies for the treatment of BCBM

### 5.1 Immunotherapy

Generally, there are no lymphocytes in the healthy brain parenchyma, which belongs to the immune-privileged site, but human BMs have been confirmed to have obvious T cell infiltration (103). Retrospective research revealed that more than 90% of tumor-infiltrating lymphocytes (TIL) were present in BCBM and its microenvironment (104). The density of TILs infiltrate is related to the size of peritumoral edema and survival prognosis, and CD8+ T cells can delay intracranial progression (103, 105). Taggart et al. demonstrated that in a mouse melanoma BM model, successful immunotherapy is associated with increased CD8+ T cell trafficking (106). Monoclonal antibodies, such as antibodies of programmed death-1 (PD1), programmed death ligand-1(PD-L1), or cytotoxic T-lymphocyte antigen 4 (CTLA4), can block immune checkpoints, and has showed moderate overall response rates in BM patients, especially in melanoma patients (107). The retrospective and prospective clinical trials showed that intracranial response rates were 16–25% after ipilimumab treatment in melanoma patients, however, these studies are very small in patient number (108, 109). More clinical studies are expected to confirm the role of immunotherapy in BCBM in the future.

### 5.2 Small molecule Vascular Endothelial Growth Factor Receptor tyrosine inhibitor

Anlotinib hydrochloride is a multi-target TKI drug that is administered orally and inhibits tumor angiogenesis and tumor cell growth by suppressing tumor-related kinases, such as TKI receptor, VEGFR 1 to 3, fibroblast growth factor receptor 1 to 4, EGFR, platelet-derived growth factor receptor α, and β, and stem cell factor receptor (110, 111). In the randomized ALTER 0303 clinical trial (a phase III trial) of advanced non-small cell lung cancer patients, in those with baseline BM, the median PFS was 4.17 months for anlotinib treatment than 1.3 months for placebo treatment (HR=0.72; 95% CI 0.15-0.56), and the median OS after anlotinib therapy was 8.57 months than 4.55 months observed after placebo therapy (HR=0.72; 95% CI 0.42–1.12) (112). Apatinib similar to anlotinib, is a small molecule that taken orally and selectively targets VEGFR-2 (113). In a phase II PATHER2 single-arm research on patients with non-small cell lung cancer, in those with baseline BM patients (n=13), the ORR was 53.8% (95% CI, 25.1-80.8%), and the median PFS was 6.7 (95% CI, 4.1-9.7) months (113). Anlotinib and apatinib have shown good efficacy in BMs of lung cancer, and more clinical data are expected to verify these efficacies in BCBM.

### 5.3 Novel therapy

In addition, there are many novel therapies being explored, including the phosphoinositide 3-kinase (PI3K)/Akt/mTOR pathway inhibitors, fatty acid synthase (FASN) inhibitors and the drugs with new delivery systems.

The PI3K/Akt/mTOR pathway is a key intracellular signaling pathway that activates tyrosine kinase receptors or G protein-coupled receptors through extracellular signals. It promotes many physiological processes such as survival, proliferation, metabolism, and angiogenesis (114). About 43-70% of BCBM patients have mutations in this pathway (115). In a mouse model of extensively metastasized HER2+ BC, buparlisib, an oral pan-PI3K inhibitor, effectively controlled the metastasis in various organs, including the brain (116). Alpelisib is another PI3K inhibition, which is reported to be effective in BCBM in some case reports (117). Ipatasertib, one of the selective ATP-competitive Akt inhibitors, shows an effective response in preclinical BCBM models (115). The mTOR, a downstream effector of the PI3K-Akt pathway, has been indicated to modulate PI3K inhibition resistance in BC, whereas, the combination of PI3K and mTOR inhibitors can overcome this resistance in a HER2+ BCBM model (118). Gdc-0084 is a dual PI3K/mTOR inhibitor with brain permeability, which has been proven to significantly inhibit PIK3CA mutant tumor growth in a patient-derived BM mouse model (119). Relevant clinical trials are underway.

The brain tissues are deficient in several nutrients necessary for cancer cells. Ferraro et al. found up-regulated fatty acid synthesis genes in BMs with a mouse model, whereas no effect was seen in in-vitro BM system, suggesting that the brain microenvironment itself promotes increased tumor fatty acid synthesis. Compared with primary breast tumors or metastasis to other sites, FASN and its encoding mRNA are highly expressed in BCBM. The use of FASN inhibitors can reduce the growth of BCBMs (120).

Since BBB may theoretically hinder the delivery of drugs to the brain, some drug delivery systems are being developed to cross BBB. One such system that is being tested is microbubble-assisted focused ultrasound (FUS), which uses oscillating microbubbles to produce micrometer-scale mechanofluidic effects to increase drug transport. When trastuzumab was combined with FUS, they revealed a potent anticancer activity in the rat brain, and also increased survival significantly (121). Another system being investigated is nanoparticles conjugated anticancer agents (122). Patil et al. demonstrated that the EGFR or HER2 inhibitors carried by nano-conjugates markedly prolong the survival of mice with HER2+ BCBM (122). Hamilton et al. also revealed that tumor-penetrating peptides coated nanoparticles can block tumor progression when tested in a preclinical BCBM model (123).

Furthermore, except the potential strategies mentioned above, there are a lot of novel treatments, such as dendritic cell vaccines, chimeric antigen receptor T-cell therapy, and so on. **Table 3** reveals the ongoing clinical trials of BCBM. In order to improve the efficacy, new therapeutic strategies are the combinations of immunotherapy, radiotherapy and targeted therapies, and the corresponding side effects also follow. We look forward to having a treatment scheme that is most beneficial to patients among the controllable side effects.

**Table 3:** Ongoing clinical trials for metastatic breast cancer with brain metastases.

Treatment	ClinicalTrials.gov	Phase	Patients’ Population	Primary Endpoint
Neratinib + capecitabine	NCT04965064	II	HER2- BCBM and abnormally active HER2 signaling	OS, CNS-PFS
Pyrotinib + vinorelbine	NCT03933982	II	HER2+ BCBM	CNS-ORR
Palbociclib + trastuzumab + pyrotinib + fulvestrant	NCT04334330	II	HR+/HER2+ BCBM	CNS-ORR
Pyrotinib + trastuzumab + abraxane	NCT04639271	II	HER2+ BCBM	CNS-ORR, CNS-PFS
T-DXd	NCT04752059	II	HER2+ BCBM	CNS-ORR
T-DXd	NCT04739761	III	Advanced or metastatic HER2+ BC	ORR; PFS
GDC-0084 + trastuzumab	NCT03765983	II	HER2+ BCBM	CNS-ORR
Trastuzumab + taxanes + pertuzumab vs. trastuzumab + taxanes + TKIs	NCT04760431	II	HER2+ BCBM	CNS-ORR
ARX788	NCT05018702	II	HER2+ BCBM	CNS clinical benefit rate
T-DM1 + afatinib vs. T-DM1	NCT04158947	II	Active refractory HER2+ BCBM	Safety and tolerability of T-DM1 and afatinib; ORR
Trastuzumab/pertuzumab + tucatinib or T-DM1 + tucatinib	NCT05323955	II	HER2+ BCBM	PFS
Phase I: T-DM1 + TMZ in dose escalation Phase II: T-DM1 vs. T-DM1 + TMZ	NCT03190967	I/II	HER2+ BCBM following SRS	MTD of temozolomide when used with T-DM; Median amount of time subject survives without disease progression after treatment.
Pyrotinib + capecitabine + brain radiotherapy	NCT04582968	I/II	HER2+ BCBM	Assess safety and tolerability (Phase Ib part); Intracranial local tumor control rate (Phase II part)
SRT + pyrotinib + capecitabine vs. WBRT + pyrotinib + capecitabine	NCT05042791	II	HER2+ BCBM	CNS-ORR
Abemaciclib + elacestrant	NCT04791384	Ib/II	HR+/HER2+ BCBM	Adverse events; iORR
Utidelone + bevacizumab	NCT05357417	II	BCBM	CNS-ORR
Nivolumab + SRS	NCT03807765	Ib	BCBM	Number of participants who experience dose limiting toxicities
Abemaciclib + SRT	NCT04923542	I/II	HR+/HER2- BCBM	CNS-PFS
Cycle 1: Olaparib + SRS Cycle 2 and 2+: Physician’s choice systemic therapy and durvalumab	NCT04711824	I/II	TN or BRCA-mutated BCBM	Frequency and severity of adverse events; intracranial disease control rate
Liposomal irinotecan + pembrolizumab	NCT05255666	II	TN BCBM	CNS disease control rate
Nal-IRI	NCT03328884	II	Progressing HER2- BCBM	CNS-ORR
QBS72S	NCT05305365	IIa	TN BCBM	CNS-ORR
ANG1005	NCT02048059	II	Recurrent BCBM	iORR
Bintrafusp alfa + pimasertib	NCT04789668	I/II	BM	Clinical benefit rate; toxicities and dose-limiting toxicities; time to intracranial progression; OS
Anti-HER2/3 dendritic cell vaccine + pembrolizumab	NCT04348747	II	TN or HER2+ BCBM	CNS-ORR
HER2-CAR T cells	NCT03696030	I	HER2+ BCBM	Incidence of dose limiting toxicities; number of participants with treatment related adverse events

BC, breast cancer; BCBM, breast cancer brain metastases; BM, brain metastasis; CNS, central nervous system; CAR, Chimeric Antigen Receptor; HR, hormone receptor; HER2, human epidermal growth factor receptor 2; iORR, intracranial objective response rate; nal-IRI, nanoliposomal irinotecan; OS, overall survival; ORR, objective response rate; PFS, progressionfree survival; SRS, stereotactic radiosurgery; SRT, stereotactic radiotherapy; T-DM1, trastuzumab emtansine; T-DXd, trastuzumab deruxtecan; TN, triple negative; TTP, time to progression; WBRT, whole brain radiotherapy.

## 6 Future prospects

In the future, compared with the systemic treatment of patients with BM, prevention of BM from the primary tumors seems to be a more important clinical goal. How to accurately screen the high-risk population with BM from BC patients requires more clinical research to explore a reliable prediction model. With the rise of liquid biopsy technology represented by circulating tumor cells (CTCs), it provides strong support for the detection of circulating brain-tropic cancer cells before their extravasation (114). The gene signature of CTCs associated with BCBM has revealed the up-regulation of Notch signaling (124). Notch targeted therapy may specifically reduce the incidence of BM in BC.

## 7 Conclusions

BM is an important clinical source of morbidity and mortality in patients with metastatic BC (43). Local interventions are the pillar of BM management (19). Systemic treatments are often used to complement local strategies to achieve optimal control of CNS diseases (19). At present, with the emergence of various new drugs, some systematic therapies have shown promising clinical results. The best results published to date have been obtained in patients with HER2+ BCBM, especially in new BM, for whom currently used combinations of chemotherapy and anti-HER2 therapy have shown certain efficacy, with particularly impressive results obtained with pirotinib + capecitabine and trastuzumab deruxtecan. However, patients with BM from HR+ BC or TNBC lack effective medical options currently, PI3K inhibitors, FASN inhibitors and immunotherapy are promising therapeutic candidates. In the coming years, the results of ongoing clinical trials with combinations of immunotherapy, radiotherapy and targeted therapies may provide better treatment options for BCBM patients.

## Author contributions

Conceptualization, YZ. Writing – original draft, QC and JX. Writing – review and editing. QC, JX, YM and JW. Supervision, CL and YZ. All authors contributed to the article and approved the submitted version.
